# Functional and Taxonomic Differentiation of Macrophyte Assemblages Across the Yangtze River Floodplain Under Human Impacts

**DOI:** 10.3389/fpls.2018.00387

**Published:** 2018-03-27

**Authors:** Min Zhang, Jorge García Molinos, Xiaolin Zhang, Jun Xu

**Affiliations:** ^1^College of Fisheries, Huazhong Agricultural University, Hubei Provincial Engineering Laboratory for Pond Aquaculture, Freshwater Aquaculture Collaborative Innovation Center of Hubei Province, Wuhan, China; ^2^Arctic Research Center, Hokkaido University, Sapporo, Japan; ^3^Global Station for Arctic Research, Global Institution for Collaborative Research and Education, Hokkaido University, Sapporo, Japan; ^4^Institute of Hydrobiology, Chinese Academy of Sciences, Wuhan, China

**Keywords:** beta-diversity, freshwater macrophyte, functional diversity, functional richness, taxonomic dissimilarity, historical change

## Abstract

Human activities and the consequent extirpations of species have been changing the composition of species assemblages worldwide. These anthropogenic impacts alter not only the richness of assemblages but also the biological dissimilarity among them. One of the main gaps in the assessment of biodiversity change in freshwater ecosystems is our limited understanding regarding how taxonomic and functional facets of macrophyte assemblages respond to human impacts on regional scales. Here, we assess the temporal (before 1970s against after 2000s) changes in taxonomic and functional richness and compositional dissimilarities, partitioned into its turnover and nestedness components, of freshwater macrophyte assemblages across the floodplain lakes of the Yangtze River in China. We found that functional and taxonomic assemblage differentiation occurred simultaneously under increasing human impact, concomitant to a general decrease in functional and taxonomic richness. However, this effect weakened when the historical level of taxonomic dissimilarity among assemblages was high. Macrophyte species with large dispersal range and submersed life form were significantly more susceptible to extirpation. The impact of human activities on differentiation was complex but habitat loss and fishery intensity were consistently the main drivers of assemblage change in these lakes, whereas water quality (i.e., light pollution and nutrient enrichment) had weaker effects. Further, macrophyte taxonomic and functional differentiation was mainly driven by the nestedness component of dissimilarity, accounting for changes in assemblage composition related to changes in species richness independent of species replacement. This result, markedly different from previous studies on freshwater fish assemblages conducted in these lakes, represents a novel contribution toward achieving a more holistic understanding of how human impacts contribute to shape community assemblages in natural ecosystems.

## Introduction

Biodiversity loss is strongly correlated with human activity because growth and spatial expansion of human population is inevitably accompanied by changes in land use, pollution and exploitation of natural resources (Vorosmarty et al., 2010; Su et al., 2015). In response to the growing threat of global biodiversity loss, much effort has been devoted to exploring and predicting the consequences of anthropogenic disturbance in ecological communities. Taxonomically, human-driven global or regional change over the last centuries has deeply reduced species richness and levels of endemism, and decreased spatial turnover across scales (Strecker et al., 2011; Villéger et al., 2014; Su et al., 2015). However, a growing body of evidence indicates that the effects of alterations in the number and identity of species comprising a community on ecosystem functioning are generally weak compared to those generated by changes in their functional diversity (Petchey and Gaston, 2002; Gacia et al., 2009; Cadotte et al., 2011; Su et al., 2015). From the perspective of biodiversity conservation, both taxonomic and functional diversity should be considered for ensuring the provision of ecosystem services (Vorosmarty et al., 2010; Su et al., 2015).

**FIGURE 1:**
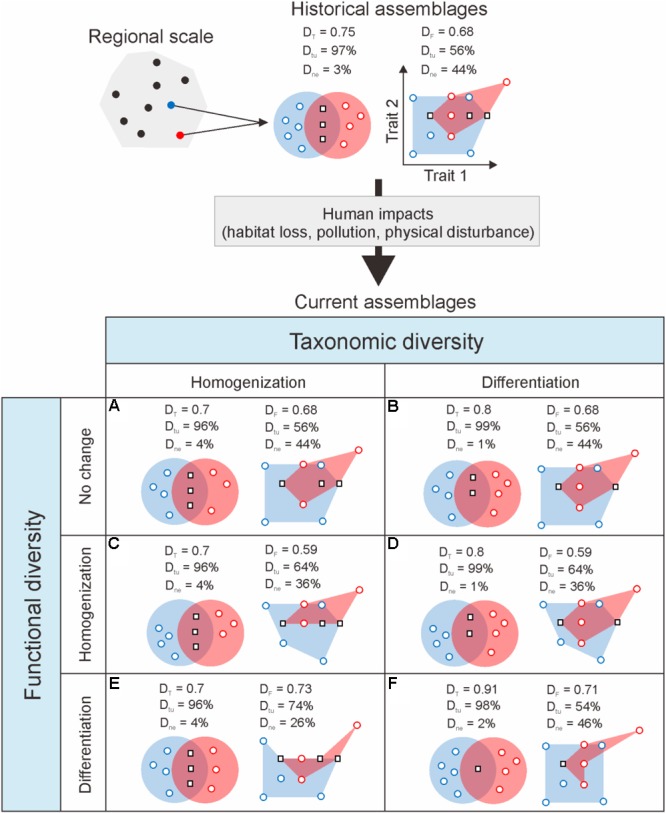
Schematic of possible temporal changes in taxonomic (*D*_T_) and functional (*D*_F_) dissimilarity between a pair of theoretical species assemblages resulting from the loss of species under human impact (adapted from Villéger et al., 2014). By modifying existing environmental conditions, resource availability, biotic interactions and dispersal processes, human activities are expected to alter the taxonomic and functional composition of assemblages. For simplicity, here those effects are represented as the loss of two species from the original assemblages comprising eight and six species of which three are common to both assemblages. Species assemblages are represented by the red and blue envelopes containing the individual species symbolized by circles (species unique to one assemblage) and squares (shared species). Taxonomic and functional similarity are, respectively, illustrated by the intersection of ellipses, following the classical Venn diagram representation, and minimum convex hulls including all species in an assemblage in a multidimensional functional space (here represented by two traits). Taxonomic homogenization **(A,C,E)** results from the loss of unique species. Conversely, taxonomic differentiation **(B,D,F)** occurs following the loss of shared species from the assemblages. Depending on the functional role of the species lost, changes in taxonomic diversity may lead to changes in functional diversity of the same sign **(C,F)**, the opposite sign **(D,E)**, or produce no change **(A,B)**. Where immigration through dispersal is possible, the arrival of new species from other assemblages may buffer or exacerbate these effects. Changes in dissimilarity can be decomposed into two complementary components related to differences in species richness between assemblages (nestedness; *D*_ne_), and the number of species replaced between assemblages independent of differences in species richness (turnover; *D*_tu_).

In nature, the local environment acts as a filter that conditions the identity and relative abundance of species present in a community in relation to their functional traits, and their interactions under niche partitioning processes (Keddy, 1992; Maire et al., 2012; Laliberté et al., 2014). Though other mechanisms are also important (Vellend, 2010), i.e., drift, speciation, and dispersal, non-random species sorting in relation to environmental filtering is often a dominant force driving community assembly patterns under stressful environmental conditions (Chase, 2007; Laliberté et al., 2014). By modifying the environmental conditions to which species are adapted, anthropogenic disturbances are an important driver of resulting assembly patterns under environmental filtering. Depending on type and strength, human disturbances can lead to increased taxonomic and functional differentiation (greater dissimilarity) or homogenization (greater similarity) of communities across space and time. For example, increased beta diversity can result from habitat fragmentation due to limitations on dispersal (Edge et al., 2017). Similarly, land use change can promote or reduce diversity among communities (spatial turnover) depending on its effects on environmental heterogeneity (Hawkins et al., 2015). Often, reduced environmental heterogeneity promotes taxonomic and functional homogenization by favoring common, widespread species (Gámez-Virués et al., 2015). These patterns are usually pervasive across spatial scales (Eskildsen et al., 2015), though the sign and underlying mechanisms may differ (Smart et al., 2006). Hence, quantification of changes in the functional diversity of communities after human disturbance in addition to changes in taxonomic diversity is crucial for attaining better understanding of regional diversity changes (Baselga et al., 2012; Villéger et al., 2014; Su et al., 2015). Variations in taxonomic and functional beta diversity convey signals of species loss dynamics emerging from the relative loss of shared and unique species from assemblages, which can lead to the differentiation or homogenization of assemblages (**Figure 1**). This is in turn related to two independent but complementary beta diversity components: a nestedness component, accounting for changes in species richness between assemblages, and a turnover components, related to the proportion of species replaced between assemblages independent of differences in species richness (Baselga et al., 2012; Villéger et al., 2014; Su et al., 2015). Testing for the differential effects of human disturbances on these components can therefore offer insight into the mechanisms behind observed diversity changes.

Few studies have so far quantitatively tested the influence of multiple anthropogenic activities on species assemblages across spatial scales (Xu et al., 2016; Cai et al., 2017); none of them on aquatic plants. Macrophytes are key components of aquatic communities and influence many structural and functional aspects of freshwater ecosystems (Carpenter and Lodge, 1986; Sondergaard and Moss, 1998). They are highly diversified and productive communities that play an important role in carbon and nutrient fluxes, altering water and sediment quality by serving as long term sinks for organic material, and short-term sources of nutrients to the water (Sondergaard and Moss, 1998; Engelhardt and Ritchie, 2001; Smith and Schindler, 2009). Macrophytes are also a food source for many primary consumers, alter flow and wave dynamics, and influence ecosystem services such as recreational use and landscape value (Bakker et al., 2013). Importantly, changes in the distribution and biomass of macrophytes have strong management implications because, given their role in sediment retention, nutrient filtering and physical sheltering against the effects of wave action, a decline or disappearance of macrophytes is often associated with shifts to phytoplankton dominance, a turbid state which may result in light limitation (Sondergaard and Moss, 1998; Smith and Schindler, 2009; Hansson et al., 2010). Indeed, the success of lake restoration efforts is often measured in terms of the extent of return of submerged macrophytes (Engelhardt and Ritchie, 2001; Paillex et al., 2009; Bakker et al., 2013). Thus, a comprehensive analysis of changes in macrophyte diversity in relation to human drivers comparable to those recently completed for other taxa should provide valuable information for lake management and conservation.

This study contributes to fill this gap by assessing the changes in taxonomic and functional diversity of Chinese floodplain lake macrophyte assemblages exposed to multiple human disturbances. We focus our analysis on: (i) the changes in, and congruence amongst (redundancy), multiple biodiversity facets (taxonomic and functional) that have emerged at regional and local scales following the drastic intensification of human activities in these lakes (before-after comparison) (Xu et al., 2016), (ii) the sensitivity of different functional traits to human alteration, and (iii) the relative contribution of historical community dissimilarity toward the extent and type of observed responses. Given the type and magnitude of existing human impacts in these lakes (see section “Materials and Methods” for a detailed account), our initial expectation is a strong spatiotemporal homogenization of assemblages driven by the loss of unique species from the assemblages. Reduced environmental heterogeneity is expected to promote taxonomic homogenization by favoring common, widespread species. These effects have been documented in assemblages following habitat loss (Puttker et al., 2015; Villegas Vallejos et al., 2016), excessive nutrient enrichment (Donohue et al., 2009; Cook et al., 2017), and physical disturbance (Passy and Blanchet, 2007); all important impacts in our lakes. Evidence on functional trait responses to these types of disturbance is scarce though we anticipate the selection toward generalist species to be also reflected in the functional homogenization of the assemblages through promotion of traits associated with ecological generalism (e.g., high dispersal and reproductive capacity). We further hypothesize that the taxonomic and functional homogenization of assemblages under human impact should operate primarily through changes in nestedness rather than turnover (i.e., human impacts decimating historical assemblages toward nested subsets with prevalence of impact-tolerant generalists) (Gutiérrez-Cánovas et al., 2013). This effect should *a priori* be stronger where historical dissimilarity between assemblages is high (i.e., higher numbers of unique non-shared species).

## Materials and Methods

### Study Lakes and Macrophyte Assemblages

The Yangtze River floodplain is among the most species-rich environments in China; their fluvial dynamics creating an intricated mosaic of habitats and gradients of hydrological connectivity. A vast extension (15,770 km^2^) of the mid and lower Yangtze River floodplain is covered by numerous lakes characterized by their shallow, flat, and large basins, and, in many cases, a long history of human alteration over the last six decades (Wang et al., 2016). This includes the rapid loss of lake area due to land reclamation and increasing sedimentation from deforestation, denaturalization of lake shore habitats, hydrological alteration, water pollution by increasing waste water discharge, and overexploitation of biological resources.

Historical (before 1970s) macrophyte assemblages from 30 of these floodplain lakes, were compiled based on presence/absence data from reported field surveys in the published and gray literature. Here, we use the definition of aquatic plant offered by Cook (1974), namely any plant visible to the naked eye, nearly always identifiable when observed, and ‘whose photosynthetically active parts are permanently or, at least for several months each year submerged in freshwater or floating on the water surface.’ This definition includes all higher aquatic plants, vascular cryptograms and bryophytes, together with groups of algae which can be seen to be composed predominantly of a single species. To ensure good quality of the dataset, we excluded lakes with poor sampling effort (i.e., sampled less than twice a year), and pooled the data where more than one survey was available for the same lake. This measure was taken for making the historical data set comparable with the contemporary surveys (see below). Within-year replication, typically during summer–autumn, provided also a measure of variability in species composition at the lakes within the growing season. Current macrophyte assemblages were characterized based on published and unpublished field survey data collected between 2008 and 2013. Species occurrence in the lakes was assessed on foot along the lake shore and by boat through transects. For each lake, triplicate macrophyte samples were collected by rake with hooks at 0, 0.5, and 1.0 m intervals at each of the 10–20 transects established randomly for every 20 km^2^ of lake surface area (Gunn et al., 2010). Each lake was sampled during the summer and autumn, and was visited at least twice during the studied period (i.e., a minimum of 4 surveys per lake). This procedure was assumed to control for the intra-annual variation in macrophyte distribution. To ensure consistency among historical and present-day surveys and reduce the potential bias introduced by differences in sampling methods, abundance or frequency data was converted into presence-absence data. The taxonomy of species documented from both historical and current field records was standardized using the Chinese Virtual Herbarium^1^. After excluding all non-native species, synonyms were unified and varieties treated as the same species. Though the number of invasive species found in these lakes increased from historical (4 species) to current (10 species) surveys, we decided to exclude them because of their low relative numbers and abundance, and because they are typically found on small ponds around the lakes rather than in the lakes. Recognizing that the exact replication of the conditions under which historical surveys were conducted is impossible, our efforts to keep consistency should allow comparison of the historical and present-day surveys.

### Measurements of Multiple Facets of Diversity

Taxonomic and functional richness were calculated, respectively, as the number of species comprising an assemblage and the value of the convex hull volume filled by a community in multidimensional functional space defined by the species traits (Villéger et al., 2008). Functional richness thus equals to the total branch length needed to join all species in an assemblage standardized to range between 0 (assemblages composed of 1 species) and 1 (Villéger et al., 2008). This definition of functional richness aims to quantify resource use complementarity (i.e., niche complementarity due to plasticity in resource use), and has been suggested to generally perform better as a predictor of ecosystem functioning than alternative methods (Petchey and Gaston, 2002). Functional richness was calculated based on four categorical functional traits extracted for each macrophyte species from the Flora of China. These include life form (submerged, floating-leaved, emergent, free-floating), life cycle (annual, perennial), morphology (turion, stem, rosette, leafy), dispersal range (local, regional, Asian endemic, and cosmopolitan) and sexual propagation (monoecism, dioecy). Taxonomic dissimilarity was then defined in terms of differences in species composition using the Jaccard index (Villéger et al., 2014). Finally, functional dissimilarity was estimated as the percentage of overlap in functional space defined by the intersection of the convex hulls corresponding to different assemblages (Villéger et al., 2014).

We partitioned both taxonomic (Baselga, 2012) and functional (Villéger et al., 2013) diversity indices into turnover and nestedness components to assess the relative contribution of the different processes leading to changes in assemblage composition, namely species replacement (turnover) and changes in species numbers without replacement (nestedness). At both extremes of the gradient, high dissimilarity between two assemblages can result from them sharing no species despite having equal numbers (complete turnover without nestedness), or from the assemblages having very different number of species despite sharing them; i.e., the species-poor being a subset of, or nested in, the species-rich assemblage.

### Assessment of Temporal Changes in Multiple Facets of Diversity

Temporal change in the taxonomic and functional facets of biodiversity was calculated as the change in the diversity shared by two assemblages between historical and current times following the method of Villéger et al. (2014). Compositional changes can result in a decrease in dissimilarity (i.e., an increase in the percentage of species or functional space shared), leading to the taxonomic or functional homogenization of assemblages, or an increase in dissimilarity indicating biotic differentiation. These processes of change in functional and taxonomic dissimilarity are *a priori* independent of each other. For instance, two assemblages may become more taxonomically dissimilar over time, because differences in the number or identity of their constituent species increase, yet become functionally homogenized where those species are highly functional redundant (Villéger et al., 2014).

### Human Activity Impact

Based on *a priori* knowledge of this area (Wang et al., 2016), we considered several factors expected to influence the diversity patterns of macrophytes among the study lakes including habitat loss, nutrient enrichment, light availability, and overexploitation of local fishery. (1) Habitat loss in these lakes has been severe following intense land reclamation from the 1950s to 1970s. At present total lake surface area is only 60% of what it was in the 1950s (25,828 km^2^). (2) Nutrient enrichment has been continuously ongoing since the 1970s because of the discharge of wastewater and sewage, resulting in more than 40% of the study lakes being currently in eutrophic–hypertrophic conditions. (3) Fishery activity is an important source of disturbance for macrophyte communities in these shallow lakes, which have been intensively fished for decades. Fishermen use bottom towed gears that induce major physical disturbance to littoral areas, removing or damaging macrophytes. (4) Lastly, light penetration into the water column, essential for macrophyte grow, can be reduced by the combined effect of multiple factors associated with human activities in these lakes such as phytoplankton blooms, heavy inputs of particulate and dissolved organic matter, and the resuspension of mineral particles from the sediment (Cristofor et al., 1994). River–lake disconnection has also been suggested as an important driver (Wang et al., 2016) but cannot be considered here because, except for Lakes Dongting and Poyang, disconnection of our study lakes from the Yangtze main stem by construction of embankments and sluice gates occurred before the historical data considered in this study.

Based on these expectations we used lake surface area (km^2^), total phosphorus concentration (mg L^-1^), Secchi depth (cm) and fishery annual catch (tons km^-2^) for each lake as descriptors of habitat loss, nutrient enrichment, light penetration, and fishery intensity during both the historical (before 1970s) and current (after 2000s) periods. Relationships between macrophyte assemblage metrics and each of these covariates were evaluated from the respective relative changes between periods. Data were pooled and averaged where more than one survey was available for the same lake.

### Statistical Analyses

Functional richness and dissimilarity were computed on the functional space made by the first three principal axes of a principal coordinates analysis (PCoA) conducted on the species functional distance matrix using Euclidean distance (Villéger et al., 2008). The resulting three-dimensional functional space provided an accurate representation of the functional dissimilarity between species explaining about 65% of the total functional space (see section “Results”), thus achieving the necessary trade-off between information quality and computation time (Villéger et al., 2008, 2013). Indices of taxonomic and functional richness, total dissimilarity and its partition into turnover and nestedness components between each pair of macrophyte assemblages were calculated from the resulting functional space computed for historical and current periods according to existing methodology (Villéger et al., 2008, 2014; Baselga, 2010, 2012). Temporal changes were then computed for these eight indices, where a positive value indicates an increase in the corresponding diversity index from the historical to the current period.

Based on our hypotheses, relationships between extirpation frequency and functional traits (as defined by the PCoA axes) were examined using generalized linear regression models (GLM), assuming a Poisson error distribution of the dependent variable. Relationships between the observed relative changes in taxonomic and functional alpha and beta diversity and human impacts were assessed using GLM, with a Gaussian error distribution, and multiple regression model based on distance matrices (MRM), respectively. Since the purpose of the model was explanatory and not predictive (i.e., assess the effect of human disturbances on changes in richness and diversity metrics), we used the full model including all four predictors (change in lake area, TP concentration, Secchi depth and fishery annual catch) after confirming model parameters were not affected by multicollinearity among predictors (variance inflation factors of all predictors < 2) (Zuur et al., 2010). To account for the potential spatial correlation among lakes we used the geographical distance among lake centroids as an extra random covariate in the models. All predictors were logarithmic or square root transformed as required to achieve normality and standardized to a scale between 0 and 1. Congruence between taxonomic and functional richness was assessed by mean of the Pearson’s correlation coefficient, while that between taxonomic and functional dissimilarity was tested with the Mantel’s permutation test. Mantel’s permutation test can be used to estimate the resemblance between two proximity matrices computed about the same object in a generalized linear regression approach. We also used this test to look for relationships between historical taxonomic dissimilarity and extirpation frequency with changes in taxonomic and functional dissimilarity. *P*-values (0.05 significance level) for Mantel’s permutation test and MRM models were obtained by comparing each observed regression coefficient with a distribution of 10,000 permuted values. The statistical analyses and plotting were conducted in R 3.1.0 (R Development Core Team, 2014) using the built-in functions and those in the packages reshape2 (Wickham, 2007), FD (Casanoves et al., 2011), betapart (Baselga and Orme, 2012), lme4 (Bates et al., 2011), ecodist (Goslee and Urban, 2007), vegan (Dixon, 2009), sjPlot (Lüdecke, 2015), and ggplot2 (Wickham, 2009).

## Results

The historical regional species pool for the 30 studied Yangtze River floodplain lakes (275 macrophyte species) was reduced by 13% (240 species) across the current assemblages. The taxonomic richness in these lakes has nonetheless decreased on average by 41.2% from the historical to the current period (from 67.5 ± 30.0 to 39.5 ± 32.2 species, mean ± SD; paired *t*-test, *t* = 3.01, *n* = 30, *P* = 0.004).

### Historical Patterns of Taxonomic and Functional Diversity

Overall differences in richness between historical assemblages were virtually identical for species and functional richness (minimum-to-maximum richness ratio of 0.595 and 0.597, respectively; **Table 1**). Historical taxonomic dissimilarity among macrophyte assemblages was relatively high (0.66 ± 0.13), with turnover contributing twice as much as nestedness toward total dissimilarity (**Table 1**). In contrast, historical functional dissimilarity (0.5 ± 0.2) was significantly lower than taxonomic dissimilarity, with the relative contribution of both components being around the same values as for taxonomic dissimilarity but reversed, i.e., nestedness contributing to total dissimilarity twice as much as turnover (**Table 1**).

**Table 1:** Values (mean ± SD, ranges in parentheses) of richness ratio (minimum/maximum richness for each pair of assemblages), taxonomic and functional dissimilarities and contribution of turnover to dissimilarity in historical and current periods and the temporal changes (current value relative to historical).

		Taxonomy	Function
Richness ratio	Historical	0.595 ± 0.230 (0.102,1)	0.597 ± 0.254 (0.019,1)
	Current	0.467 ± 0.292 (0.085,1)	0.344 ± 0.299 (0.003,1)
	Change	–0.128 ± 0.309 (-0.79,0.78)	–0.253 ± 0.374 (-0.961,0.851)
Dissimilarity	Historical	0.656 ± 0.126 (0.031,0.948)	0.5 ± 0.196 (0,0.981)
	Current	0.736 ± 0.135 (0.286,0.933)	0.716 ± 0.249 (0,0.997)
	Change	0.08 ± 0.165 (-0.573,0.674)	0.216 ± 0.3 (-0.667,0.85)
Contribution of turnover to dissimilarity	Historical	0.669 ± 0.244 (0,1)	0.327 ± 0.333 (0,0.998)
	Current	0.558 ± 0.321 (0,1)	0.2 ± 0.296 (0,0.998)
	Change	–0.111 ± 0.305 (-0.909,0.864)	–0.127 ± 0.419 (-0.998,0.959)
Contribution of nestedness to dissimilarity	Historical	0.331 ± 0.244 (0,1)	0.67 ± 0.335 (0,1)
	Current	0.442 ± 0.321 (0,1)	0.77 ± 0.323 (0,1)
	Change	0.111 ± 0.305 (-0.864,0.909)	0.1 ± 0.448 (-1,0.998)

### Temporal Changes in Taxonomic and Functional Diversity

The ratio of taxonomic richness between the species poorest and richest assemblages in each pair decreased between historical and current periods both for taxonomic and functional richness, though the reduction experienced in functional richness doubled that of taxonomic richness (**Table 1**). Macrophyte assemblages showed a weak trend toward taxonomic differentiation between periods (mean dissimilarity increase of 0.08 ± 0.17; **Table 2**). Cases of taxonomic homogenization (i.e., assemblages that became more similar in species composition) were clearly less frequent than those of taxonomic differentiation (26.4% vs. 73.6%; **Table 2**). Contribution of turnover to taxonomic dissimilarity decreased and that of nestedness increased by 11% (**Table 1**). These changes were mostly related to the taxonomic differentiation of assemblages (**Table 2**).

**Table 2:** Summary of frequency and intensity of changes in taxonomic dissimilarity, functional dissimilarity and contribution of functional turnover to functional dissimilarity across the study lakes.

	Taxonomic homogenization	Taxonomic differentiation	Functional homogenization	Functional differentiation
Total change	26.4% (–0.114 ± 0.114)	73.6% (0.15 ± 0.118)	25.3% (–0.2 ± 0.138)	74.7% (0.356 ± 0.189)
			TH: 16.1% (–0.242 ± 0.138)	TH: 10.3% (0.25 ± 0.183)
			TD: 9.2% (–0.127 ± 0.105)	TD: 64.4% (0.373 ± 0.185)
Decrease in contribution of turnover	9.7% (–0.169 ± 0.132)	55.4% (–0.296 ± 0.215)	10.6% (–0.37 ± 0.256)	43.4% (–0.428 ± 0.287)

			TH: 5.7% (–0.386 ± 0.292)	TH: 6.7% (–0.368 ± 0.241)
			TD: 4.8% (–0.351 ± 0.21)	TD: 36.8% (–0.439 ± 0.294)
Increase in contribution of turnover	16.8% (0.204 ± 0.208)	18.2% (0.189 ± 0.191)	9.2% (0.431 ± 0.296)	17.2% (0.336 ± 0.253)

			TH: 6.2% (0.44 ± 0.311)	TH: 3.7% (0.346 ± 0.239)
			TD: 3% (0.412 ± 0.274)	TD: 13.6% (0.333 ± 0.259)
Decrease in contribution of nestedness	16.8% (–0.204 ± 0.208)	17.2% (0.199 ± 0.191)	12.2% (–0.514 ± 0.326)	17% (–0.34 ± 0.252)

			TH: 8.5% (–0.531 ± 0.329)	TH: 3.7% (–0.346 ± 0.239)
			TD: 3.7% (–0.474 ± 0.323)	TD: 13.3% (–0.339 ± 0.258)
Increase in contribution of nestedness	9.7% (0.169 ± 0.132)	56.3% (–0.291 ± 0.217)	8.5% (0.378 ± 0.251)	43.7% (0.431 ± 0.289)

			TH: 3.9% (0.436 ± 0.303)	TH: 6.7% (0.368 ± 0.241)
			TD: 4.6% (0.33 ± 0.191)	TD: 37% (0.442 ± 0.296)

*Values in parentheses are mean ± standard deviation. TH and TD represent the fraction of assemblage pairs experiencing functional homogenization or differentiation that also developed taxonomic homogenization or differentiation.*

Change in functional dissimilarity was on average higher than change in taxonomic dissimilarity (0.216 ± 0.300; **Table 1**). However, variability was high with 74.7% of assemblage pairs showing differentiation while 25.3% showed no change or homogenization (**Table 2**). The contribution of nestedness and turnover to functional dissimilarity showed similar changes to those of taxonomic dissimilarity (**Table 1**). Among current communities, turnover was on average the major component contributing toward taxonomic and functional total beta diversity. This relative importance reflected that of historical communities for taxonomic diversity but not for functional diversity; nestedness contributing historically twice as much as turnover (**Table 1**). Among the 74.7 and 25.3% of assemblage pairs that showed functional differentiation and homogenization, respectively, a higher percentage showed a decrease in the contribution of turnover (43.4 and 10.6%) than an increase (17.2 and 9.2%) (**Table 2**).

**FIGURE 2:**
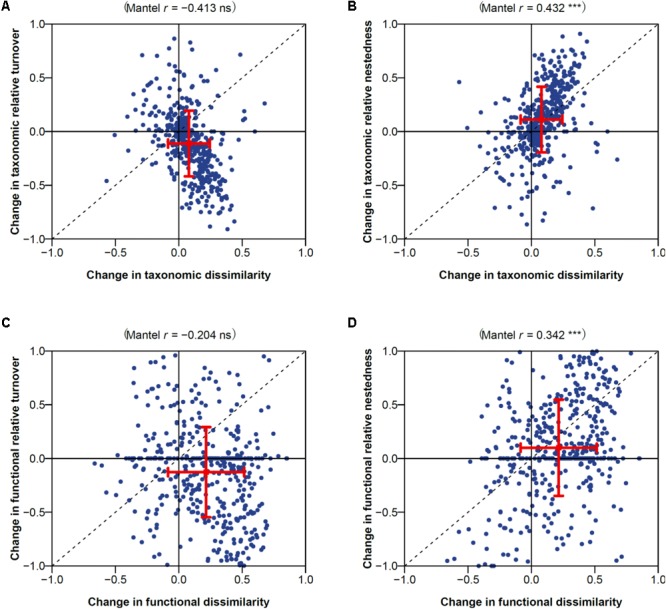
Changes in **(A,B)** taxonomic and **(C,D)** functional dissimilarity vs. changes in contribution of **(A,C)** turnover and **(B,D)** nestedness to dissimilarity. Spearman’s correlation coefficient and associated Mantel permutation test are provided at the top of each panel (^∗∗∗^*P* < 0.001; ns, not significant). The mean value and associated standard deviation among macrophyte assemblage pairs is shown in each panel.

**FIGURE 3:**
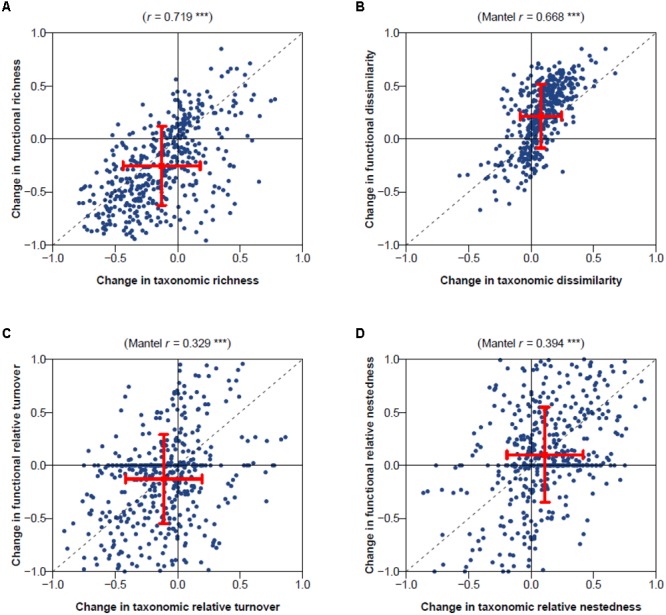
Comparison of changes in taxonomic and functional diversity for **(A)** richness, **(B)** total dissimilarity, and its **(C)** turnover and **(D)** nested components. Pearson’s correlation coefficient and associated simple linear regression test is provided at the top of the richness panel (**A**; ^∗∗∗^*P* < 0.001). Spearman’s correlation coefficient and associated Mantel permutation test are provided at the top of other panels (**B–D**; ^∗∗∗^*P* < 0.001).

Temporal changes in taxonomic and functional dissimilarity were significantly positively correlated with change in contribution of nestedness to dissimilarity (Spearman’s correlation coefficient 0.43 and 0.34; Mantel test *P* < 0.001; **Figures 2B,D**). No significant correlation was found between temporal changes in functional or taxonomic dissimilarity and changes in the contribution of turnover (**Figures 3A,C**). Highly significant positive correlations were also found between temporal changes in taxonomic and functional richness (0.72; **Figure 3A**), and total dissimilarity (0.67; **Figure 3B**). These changes were also significantly correlated to changes in the contribution of turnover and nestedness to taxonomic and functional dissimilarities between periods (**Figures 3C,D**).

### Determinants of Changes in Taxonomic and Functional Diversity

**FIGURE 4:**
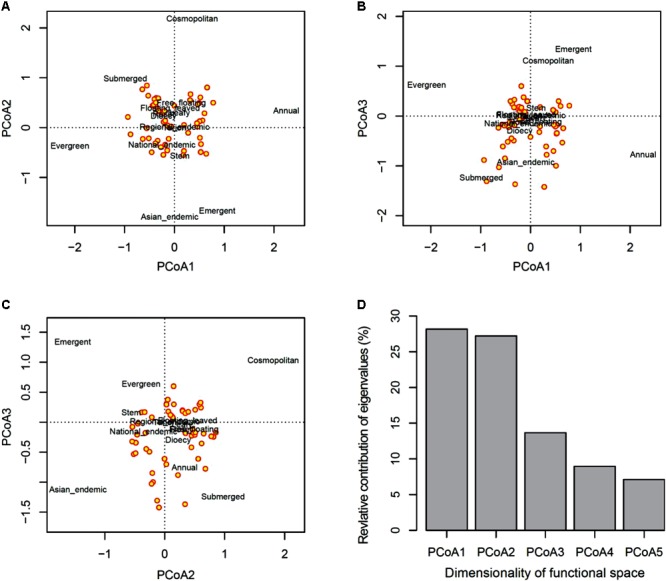
Principal coordinates analysis (PCoA) of functional traits. Shown are **(A–C)** plots of assemblage pairwise comparison of the first three PCoA axes (factors) and **(D)** the relative contribution of eigenvalues of the first five PCoA axes.

**FIGURE 5:**
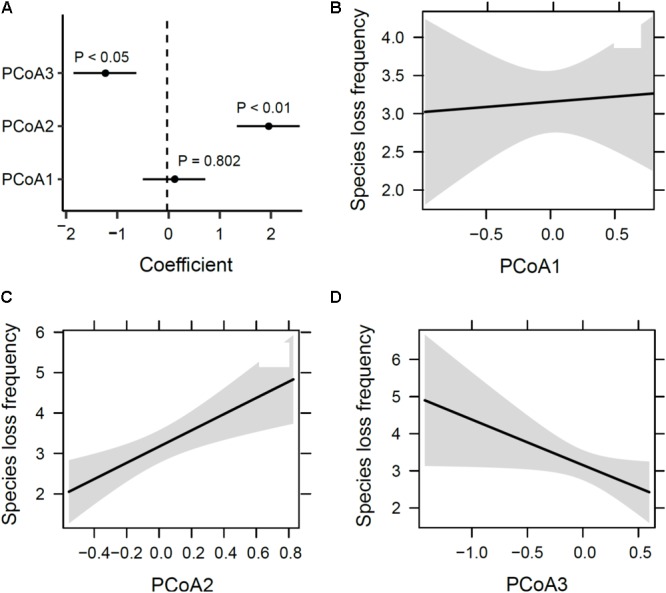
Sensitivity of species loss to functional traits. **(A)** Correlation coefficients and their significance from a generalized linear model and panels, and **(B–D)** estimated marginal effects and confidence intervals of the first three PCoA axes (see **Figure 3**).

The first three axes from the PCoA analysis of macrophyte functional traits (**Figures 4A–C**) mainly represent differences in life cycles following a low-to-high value gradient from long to short time (PcoA1), dispersal range from Asian endemic to cosmopolitan (PcoA2), and life form from underwater to above the water surface (PcoA3). This three-dimensional functional space provided an accurate representation of the functional dissimilarity between species accounting for about 65% of the total variability in trait space (**Figure 4D**). Species loss frequency was significantly related to PcoA2 and PcoA3 (**Figure 5**), with species with large-scale dispersal and submersed life from being most sensitive to extirpation.

**FIGURE 6:**
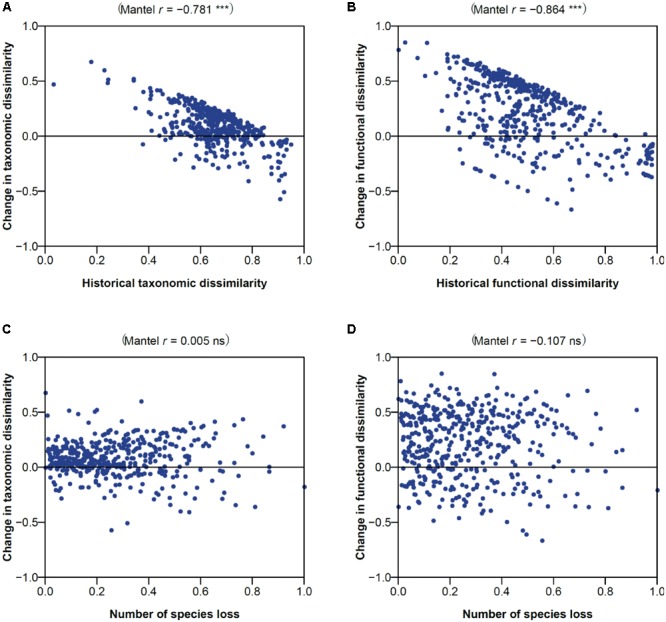
Effects of **(A,B)** historical dissimilarity and **(C,D)** species loss on the changes in **(A,C)** taxonomic and **(B,D)** functional dissimilarity. Spearman’s correlation coefficient and associated Mantel permutation test are provided at the top of each panel (*P* < 0.001; ns, not significant).

Changes in taxonomic and functional dissimilarities showed a significant negative correlation with their historical respective dissimilarities (-0.781 and -0.864; *P* < 0.001; **Figures 6A,B**), indicating a tendency for assemblage pairs exhibiting low and high historical dissimilarity to become more differentiated and homogenized over time, respectively. Changes in the taxonomic and functional dissimilarities were, however, not related with how many species were lost from assemblage pairs (**Figures 6C,D**).

**FIGURE 7:**
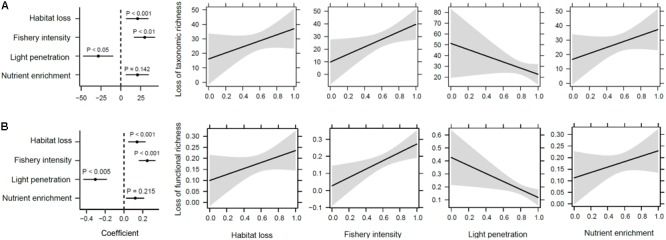
Human impacts on changes of macrophyte richness. **(A)** Taxonomic and **(B)** functional richness patterns showing from left to right the correlation coefficients and their significance from a generalized linear model, and the estimated marginal effects and confidence intervals of human impacts (i.e., habitat loss, fishery intensity, light penetration, and nutrient enrichment in sequence).

**FIGURE 8:**
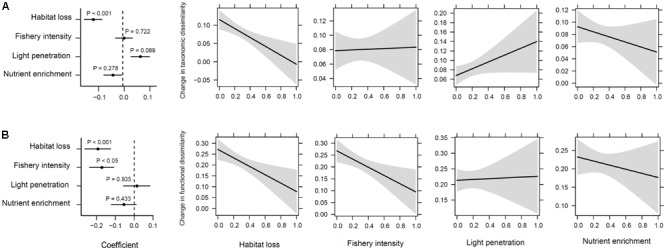
Human impacts on changes of taxonomic and functional dissimilarity. **(A)** Taxonomic dissimilarity, and **(B)** functional dissimilarity showing from left to right the correlation coefficients and their significance from a permuted multiple regression on distance matrices, and the estimated marginal effects and confidence intervals of human impacts (i.e., habitat loss, fishery intensity, light penetration, and nutrient enrichment in sequence).

Reductions in taxonomic and functional richness were significantly related to increasing habitat loss and fishery intensity, and reduced light penetration conditions (*R*^2^ = 0.579 and 0.659, respectively, *P* < 0.001; **Figures 7A,B**). Changes in total taxonomic dissimilarity were also significantly altered by human impacts (*P* < 0.001; **Figure 8A**). Contrary to richness, habitat loss had a significant negative effect on total taxonomic dissimilarity, suggesting that intense habitat loss facilitates taxonomic homogenization between pairs of lake assemblages. On the other hand, factors that influenced significantly changes in functional dissimilarity differed slightly from those that did for taxonomic dissimilarity (**Figure 8B**). Significant functional homogenization (i.e., decreased dissimilarity) resulted from assemblage pairs that experienced higher habitat loss as well as fishery intensity.

## Discussion

### Historical Diversity Patterns

The broad distributional ranges of macrophytes, traditionally explained by their long-distance anemochory, hydrochory, and zoochory seed dispersal, have been long recognized (Santamaria, 2002; Chambers et al., 2008). This pattern is also reflected in our analyses where 65% of the macrophytes studied are cosmopolitan and a further 25% are Asian endemic species, while regional and local endemic species account for just 10% of the species regional pool. Meanwhile, a strong dispersal capacity has facilitated the evolution of broad ecological tolerances and plastic responses to local environmental change in macrophytes. For instance, most macrophyte species are highly resilient because of traits such as clonal growth and the abundance of easily dislodged propagules (Santamaria, 2002; Chambers et al., 2008). Consequently, regional taxonomic richness could be expected to be high and taxonomic assemblage dissimilarity low under natural conditions. However, this outcome is not supported by our results, where the high historical level of taxonomic dissimilarity between macrophyte assemblages was mainly associated to the effect of species replacement among assemblages, as indicated by the high relative contribution of turnover to historical taxonomic dissimilarity (**Table 1**). A pattern that agrees with those reported for other aquatic plant assemblages at regional scales (Viana et al., 2015).

White et al. (2006) suggested that large temporal fluctuations in the environment might cause species to be more temporally patchy in their occurrence, which should increase species turnover. Riverine floodplains are typically highly fluctuating environments, their fluvial dynamics creating a complex mosaic of habitats and gradients of hydrological connectivity (Friedman and Auble, 2000; Paillex et al., 2009). In a historically intense hydrological disturbance environment like ours (Wang et al., 2016), the observed low richness ratio of local assemblage to regional species pool (∼24%), high taxonomic dissimilarity, and high contribution of turnover could be explained by the strong filtering effect that high environmental heterogeneity can exert on species selection and persistence. Therefore, environmental filtering can be considered the dominant driver of species turnover of macrophytes in these shallow lakes.

### Temporal Changes of Diversity

Though the direction and intensity of change in taxonomic dissimilarity among macrophyte assemblages was variable, we found a distinct overall trend toward taxonomic and functional differentiation (**Table 1** and **Figure 2**). This result is against our initial expectation of a strong biotic (taxonomic and functional) homogenization under increasing human impact. The effects of environmental filtering on biodiversity relate to the degree of adaptation of species to the local environmental conditions. Natural environmental variation is expected to result in increased species turnover, promoted by a high degree of specificity and specialism (i.e., adaptation over evolutionary time). In contrast, human disturbances should lead to the loss of species, starting by specialist taxa, where they push local environmental conditions beyond the bounds of natural variability those species are locally adapted. This should facilitate increasingly nested assemblages by reducing (subsetting) natural assemblages through filtering toward stress-tolerant generalist species (Gutiérrez-Cánovas et al., 2013). Instead, we found that the overall increasing nestedness resulting in the observed compositional assemblage differentiation was mainly driven by the selective loss of common, shared species from historical assemblages; a scenario that conforms to **Figure 1F**. This pattern does not seem to be attributable to a purely stochastic effect, whereby a decreased regional species pool effectively reduces the probability of turnover, hence artificially increasing nestedness (Chase and Myers, 2011). The nestedness component (0.221 ± 0.001) based on 9999 Monte Carlo simulations of current lake assemblages preserving the characteristics of the observed site × species matrix, following the loss of 13% of the species from the historical regional pool, was significantly lower (*P* < 0.001) than the observed mean nestedness value (0.331 ± 0.283).

Historically abundant plants shared among these lakes such as *Najas ancistrocarpa, N. graminea, Potamogeton pusillus, P. gramineus, Ottelia acuminata*, and species from genus *Blyxa* and *Batrachium*, became rare or disappeared from many of the lakes. This increased in turn the relative abundance and shifted dominance toward species already existing in the lakes such as *P. maackianus, Vallisneria denseserrulata, Hydrilla verticillata, V. natans*, or *Nymphaea tetragona* in moderately disturbed lakes, and *P. crispus, Myriophyllum spicatum, Ceratophyllum demersum, Trapa bispinosa*, or *Hydrocharis dubia* in highly disturbed lakes. This outcome seems also not attributable to a complete reorganization of the metacommunity given the observed positive correlation between historical and current dissimilarity. However, all the extirpated common species are wide-ranged littoral species with submerged life form, whereas emergent and floating-leaved plants prevail among the new dominant species. The significant and marginal relationships found between species loss and PcoA2 and PcoA3 axes confirm this relationship of cosmopolitan species with submersed life from being more susceptible to extirpation. Littoral habitats represent the ‘ecosystem frontline’ receiving the full impact from the intense land transformation to which these lakes have been exposed. Further, light attenuation from algal growth under chronic nutrient enrichment and the increasing resuspension of sediments from physical alteration of the lake bed (e.g., fishing) can have a strong inhibitory effect on submerged plants (Kemp et al., 1983). These results may be indicative of an ongoing regime shift in these lakes toward an alternative stable state with emergent and floating-plant dominance. Dense mats of floating plants can create dark, anoxic conditions that strongly reduce underwater plant and animal biomass and diversity; effects that are more pervasive under nutrient enrichment (Scheffer et al., 2003).

The significant correlation found between macrophyte taxonomic and functional diversity in these lakes (**Figure 3**) is in line with other studies that have found high congruence among spatial patterns of taxonomic and functional diversity of freshwater assemblages such as fish (Strecker et al., 2011; Pool et al., 2014) or macroinvertebrates (Heino et al., 2008). Though context dependent, this evidence makes the case that focusing on multiple facets of biodiversity may always not be as necessary as it is commonly perceived (Strecker et al., 2011; Pool et al., 2014). These results are therefore important for optimizing conservation planning, often limited by resources and priorities (Heino, 2010).

Taxonomic and functional differentiation between past and current assemblages followed complex patterns, but habitat loss and fishery intensity were consistently the main drivers of assemblage change, whereas light condition and nutrient enrichment had weaker effects. Habitat loss via residential development of littoral areas to agricultural lands, urban or suburban lands, and roads can cause not only the immediate extinction of species particular to those areas but also the delayed extinction of poorly dispersing, perhaps competitively superior, species of extant ecosystems (Tilman and Lehman, 2001). Macrophytes, in particular, are sensitive to the effects of habitat loss and fragmentation (Bakker et al., 2013). The observed decrease in total taxonomic and functional richness, together with the increasing contribution of nestedness, suggests that common submersed species are substantially more sensitive to habitat loss. Because littoral areas have higher propagule densities compared the non-littoral parts of a lake, and proportions as high as 60% of the individuals recolonizing freshwater littoral areas may originate from *in situ* vegetative propagules (Hansson et al., 2010; Bakker et al., 2013), the absence of these source of propagules due to habitat loss may further inhibit macrophyte recovery and promote higher contribution of nestedness to taxonomic diversity over time because of the loss of common species across the lakes.

Fishery activity is another important factor affecting the macrophyte diversity at local and regional scales. Stock-enhanced capture and intensive aquaculture in these lakes after 1960s has intensified following the sustainable development of lake fisheries (Jia et al., 2013). As nets, beams, trawl doors, chains and dredges pass over the lake bed, the sediment is disturbed and a large amount of the resident biota (e.g., macrophytes, snails, and bivalves) is damaged or removed (Olsgard et al., 2008; Sciberras et al., 2016). In addition to direct changes to macrophyte communities, fishery can also alter the biogeochemical characteristics of the sediment and that of the overlying water column through a combination of the removal of surficial sediments and the burial or mixing of organic matter (Olsgard et al., 2008; Sciberras et al., 2016). Indirectly, these processes increase the water turbidity and the concentration of particulate organic matter in the overlying water and may enhance phytoplankton primary production due to higher nutrient loads, which affect the diversity of macrophyte communities (Carpenter and Lodge, 1986; Hilt et al., 2013).

Light penetration only significantly decreased taxonomic and functional richness, while nutrient enrichment did not have any significant effect on macrophyte richness and diversity patterns. Lake eutrophication is one of the severe impacts of excessive wastewater and sewage in our studied region and more than 40% of the lakes were in eutrophic–hypertrophic states (Wang et al., 2016). Eutrophication commonly facilitates development of phytoplankton, which reduce light penetration and enhance competition with other primary producers, inducing the local decline or disappearance of macrophytes (Smith and Schindler, 2009; Hansson et al., 2010; Bakker et al., 2013). This effect is, however, less apparent at the lake scale, especially for large lake assemblages like ours (Smith and Schindler, 2009; Hansson et al., 2010; Bakker et al., 2013). Light penetration can also be affected by wind, fishery activity, and hydrological regime in our studied lakes. A decrease of water transparency can result from resuspended sediment particles and internal regeneration of the nutrient pool, negatively affecting macrophyte colonization (Beck et al., 2013; Hilt et al., 2013; Vila-Costa et al., 2016).

## Conclusion

Current macrophyte assemblages in these shallow floodplain lakes have experienced widespread extirpation of species relative to their historical status, resulting in the seemingly non-stochastic decline of regional species richness. These results conform to our initial expectations given the type and magnitude of existing human impacts in these lakes. Nonetheless, species losses have promoted the taxonomic and functional differentiation of the assemblages over time via increased nestedness (though turnover remained as the main contributor toward taxonomic dissimilarity). This intriguing result deviates from our expectation of a strong temporal homogenization of assemblages under increasing human impact, and originates from the selective loss of common (shared) species with submerged life form from historical assemblages with subsequent facilitation of different emergent and floating-leaved plants in the current assemblages. Our study therefore presents novel evidence on the regional patterns of taxonomic and functional diversity in macrophyte lake assemblages and how these have evolved over time in relation to the effects of developing human activities. At a regional scale, our results can be important for conservation and rehabilitation of degraded macrophyte communities under changing human-driven environmental conditions.

## Author Contributions

JX and MZ conceived the study. MZ, JX, and XZ compiled the data. MZ and JX analyzed the data. MZ, JGM, and JX wrote the paper. All authors contributed to the final manuscript.

## Conflict of Interest Statement

The authors declare that the research was conducted in the absence of any commercial or financial relationships that could be construed as a potential conflict of interest.

## Footnotes

1 http://www.cvh.ac.cn/en
